# Mechanisms underlying spontaneous and evoked calcium responses in oligodendrocyte precursor cells: A modeling investigation

**DOI:** 10.1371/journal.pcbi.1013430

**Published:** 2026-06-18

**Authors:** Martin Lardy, Leqi Wang, Claire Guerrier, Veronica T. Cheli, Pablo M. Paez, Anmar Khadra

**Affiliations:** 1 Department of Physiology, McGill University, Montreal, Quebec, Canada; 2 Department of Mathematics, McGill University, Montreal, Quebec, Canada; 3 Laboratoire Jean-Alexandre Dieudonne, Université Côte d’Azur, Nice, France; 4 Department of Pharmacology and Toxicology, University of Buffalo, Buffalo, New York, United States of America; University of Massachusetts Amherst, UNITED STATES OF AMERICA

## Abstract

Calcium (Ca^2+^) signaling has emerged as a central regulator of activity-dependent myelination in oligodendrocytes. These Ca^2+^ signals encompass both the stimulus-independent spontaneous Ca^2+^ local transients (SCaLTs) generated intrinsically in a voltage-independent manner or facilitated by the membrane voltage, as well as evoked responses triggered by ATP and glutamate release. To investigate the regulatory mechanisms underlying this combined spiking activity, we developed a stochastic spatiotemporal flux-balance model of Ca^2+^ transients in oligodendrocyte precursor cells (OPCs). The model incorporates all the relevant fluxes in these cells and integrates membrane voltage dynamics with a Ca^2+^-induced Ca^2+^-release (CICR) mechanism using parameters fitted to Ca^2+^ fluorescence recordings. The model reproduced the intrinsic and voltage-facilitated SCaLTs in OPCs in the absence of purinergic and glutamatergic receptors, and captured the three distinct patterns of evoked Ca^2+^ responses induced by prolonged ATP and glutamate stimulations identified using machine classifier. The model highlighted the role of ATP and glutamate in generating these clusters, and showed that the fast dynamics of CICR is key to producing these evoked responses. Further analysis of the model also revealed that voltage-gated L- and T-type Ca^2+^ channels slightly increase the frequency of SCaLTs, while stimulation with ATP and glutamate, using randomly distributed pulses mimicking *in vivo* conditions, leads to an increase in both the amplitudes of Ca^2+^ spikes (i.e., the combination of SCaLTs and evoked responses) and the prevalence of wide spikes, especially upon glutamate stimulation. Bifurcation analysis of the deterministic version of the model, in the absence of diffusion, demonstrated that ATP and glutamate stimulation can shift the system into an oscillatory regime, thereby increasing the deterministic component of SCaLT dynamics. This study thus offers a comprehensive representation of OPC Ca^2+^ transients linking recorded *in vitro* behaviors to *in vivo* dynamics.

## Author summary

Oligodendrocytes are glial cells in the central nervous system that form myelin, the insulating sheath enabling rapid nerve signal transmission. Myelination is a dynamic process influenced by neuronal activity, with calcium (Ca^2+^) signaling emerging as a key regulator. These signals include spontaneous local Ca^2+^ transients (SCaLTs), generated intrinsically or facilitated by membrane voltage, as well as evoked responses triggered by neurotransmitters like ATP and glutamate. To understand how these signals arise and interact, we combined experimental recordings of Ca^2+^ activity in oligodendrocyte precursor cells (OPCs) with a data-driven biophysical model. The model incorporates stochastic Ca^2+^ fluxes, membrane voltage dynamics, and Ca^2+^-induced Ca^2+^-release (CICR), allowing us to simulate diverse patterns of Ca^2+^ transients. Our simulations reproduced both intrinsic and voltage-facilitated SCaLTs and captured three distinct evoked response types induced by ATP and glutamate. We found that voltage-gated Ca^2+^ channels slightly enhance SCaLT frequency, while rapid CICR dynamics are critical for shaping the amplitude and timing of evoked signals. Furthermore, neurotransmitter stimulation can drive the system into an oscillatory regime, increasing the deterministic structure of Ca^2+^ transients. This work offers a mechanistic framework linking intracellular Ca^2+^ dynamics to the regulation of activity-dependent myelination in OPCs.

## 1 Introduction

Oligodendrocyte progenitor cells (OPCs) represent a distinctive type of stem cells within the central nervous system (CNS) of vertebrates, including humans [1]. Their significance stems from their crucial role in both the development and ongoing maintenance of the nervous system [2]. As OPCs mature, they undergo differentiation into oligodendrocytes, the cells that synthesize myelin, a lipid-rich substance, that ensheathe axons and support saltatory conduction by significantly enhancing the speed and efficiency of neural signal transmission [3–5].

The intricate interplay between axons and myelinating oligodendrocytes is fundamentally orchestrated by ion homeostasis mechanisms localized at the interface between the myelin sheath and the axons [6]. A manifestation of this intricate communication is the regulation of cytosolic calcium (Ca^2+^) concentration, denoted as [Ca^2+^]_*i*_, within oligodendrocytes [7,8]. The dynamics of [Ca^2+^]_*i*_ within the cell hold the potential to exert influence over myelin formation, remodeling, and other yet-to-be-fully-understood functions [9]. Remarkably, it was shown that myelin sheath elongation is facilitated by a high frequency Ca^2+^ transients and obstructed by buffering [10].

Spontaneous Ca^2+^ local transients (SCaLTs) have been observed in isolated OPCs [11]. These events arise from predominantly stochastic (random) processes modulated by underlying deterministic patterns [12]. SCaLTs are attributed to both intrinsic cellular mechanisms as well as to fluctuations in membrane voltage. A growing body of evidence highlights the role of key Ca^2+^ fluxes in shaping SCaLTs driven by intrinsic dynamics. Prominent among these fluxes are (i) store-operated Ca^2+^ entry (SOCE) channels that rely on the three core proteins: ORAI1, STIM1, and STIM2 to regulate Ca^2+^ influx across the cell membrane in response to changes in Ca^2+^ concentration within the endoplasmic reticulum (ER) [11,13], denoted by [Ca^2+^]_*ER*_, (ii) the sodium/calcium exchanger (NCX) that exchanges one Ca^2+^ ion for three sodium (Na^+^) ions [14], (iii) Ca^2+^ release events emanating from the ER via inositol-triphosphate receptors (IP3Rs) and Ryanodine receptors (RyRs), both of which are involved in Ca^2+^-induced Ca^2+^-release (CICR) [15,16], (iv) Ca^2+^ efflux mediated by sarco/endoplasmic reticulum Ca^2+^-ATPase (SERCA) pumps [17], and plasma membrane Ca^2+^-ATPase (PMCA) pumps [18].

The synergy of these fluxes forms the basis for SCaLTs in OPCs. Indeed, previous modeling studies of Ca^2+^ signaling in these cells have shown that fluxes through SOCE, NCX, CICR (via IP3Rs and RyRs), and PMCA and SERCA pumps are sufficient for producing intrinsic SCaLTs [12]. Interestingly, these cells also express voltage-gated Ca^2+^ channels (VGCCs), including L- and T-type Ca^2+^ channels on their membrane [19–21], which facilitates Ca^2+^ influx in response to membrane depolarization, contributing to cellular signaling and function including migration and myelination [19,21,22]. Despite their importance, it remains unclear how voltage membrane fluctuations and Ca^2+^ entry through VGCCs affect SCaLTs and their underlying dynamics.

A pivotal aspect of OPC function is their ability to receive glutamatergic signals via α-amino-3-hydroxy-5-methyl-4-isoxazolepropionic acid receptors (AMPAR) and N-methyl-D-aspartate receptors (NMDAR) [23,24]. These receptors mediate cation influx, including Ca^2+^, enabling glutamate signaling to elevate [Ca^2+^]_*i*_ either directly or indirectly [9] and to subsequently modulate subcellular processes such as proliferation and differentiation [25]. Additionally, the presence of purinergic P2X7 receptors (P2XRs) in OPCs has been implicated in diverse signaling pathways [9,26,27]. In mouse OPCs, P2X7R activation by ATP induces distinct signaling responses reminiscent of these previously analyzed [28–32], further underscoring its functional importance [9,33]. However, the interplay between purinergic and glutamatergic receptors, along with membrane depolarization, in shaping evoked Ca^2+^ responses, particularly in the context of SCaLTs, remains an open area of investigation.

In this study, we extended a previously developed stochastic spatiotemporal flux-balance model, implemented in one spatial dimension and validated against fluorescent Ca^2+^ imaging data from rat OPCs [12], to investigate the dynamics of both spontaneous and evoked Ca^2+^ transients in OPCs. The results demonstrated the model’s ability to replicate key features of evoked Ca^2+^ responses observed *in vitro* while predicting how these responses manifest *in vivo*. Specifically, it delineated the distinct effects of membrane depolarizations, glutamate signaling and ATP stimulation, with the two latter ones modeled as random processes. Additionally, the model provided novel insights into the interplay between SCaLTs and evoked responses in OPCs, and identified the role of ATP and glutamate stimulations in inducing oscillatory behaviors.

## 2 Materials and methods

### 2.1 Experimental methods

#### 2.1.1 Primary cultures of OPCs.

Primary cultures of cortical OPCs were prepared as described [34–36] which results in >98% OPCs and <1% GFAP stained astrocytes or Iba1 stained microglia. Cerebral hemispheres from 1-day old mice were mechanically dissociated, then plated in poly-D-lysine-coated flasks in DMEM/F12 (1:1 v/v) (Invitrogen) supplemented with 10% fetal bovine serum (FBS) (Life Technologies). After 4 h, the medium was changed and cells grown in DMEM/F12 supplemented with insulin (5 μg/ml), apotransferrin (50 μg/ml), Na^+^ selenite (30 nM), d-Biotin (10 mM) and 10% FBS (Life Technologies). Every 3 days 2/3 of the media was changed. OPCs were purified from mixed glia after 14 days by a differential shaking and adhesion procedure. The detached cells were plated into Petri dishes for 30 min at 37°C to allow microglia and astrocytes to adhere, then non-attached cells collected and plated on poly-D-lysine-coated coverslips in DMEM/F12 supplemented with insulin (5 μg/ml), apotransferrin (50 μg/ml), Na^+^ selenite (30 nM), 0.1% BSA, progesterone (0.06 ng/ml), putrescine (16 μg/ml) (Sigma), and 2% FBS. OPCs were kept in mitogens, platelet derived growth factor (PDGF) and basic fibroblast growth factor (bFGF, 20 ng/ml) (Peprotech), for 3 days.

#### 2.1.2 Ca^2+^ imaging.

Primary cultures of cortical OPCs were prepared as described above. Before imaging, OPCs were washed in serum and phenol-red-free DMEM and incubated for 25 min with 4 μM fura-2 (AM) (Life Technologies) plus 0.08% Pluronic F127 (Life Technologies) at 37°C and 5% CO_2_. Cells were then washed four times in DMEM and stored in DMEM for 10 min before being imaged. Ca^2+^ influx and resting Ca^2+^ levels were measured in serum and phenol-red-free HBSS containing 1.3 mM Ca^2+^ and 1 mM Mg^2+^. Fura-2 was excited by alternating 340 and 380 nm. Fluorescence signals were acquired every 2 s by means of a high-speed wavelength-switching device (Sutter Instruments, Lambda DG4). A spinning disc confocal inverted microscope (Olympus, IX83-DSU) equipped with a CCD camera (Hamamatsu, ORCA-R2) measured fluorescence. Ca^2+^ influx and resting Ca^2+^ levels were measured on individual cell bodies using the image analysis software Meta Fluor (Molecular Devices). To minimize bleaching, excitation light intensity and sampling frequency was kept as low as possible.

### 2.2 Mathematical methods

#### 2.2.1 Mathematical model.

**Fig 1:**
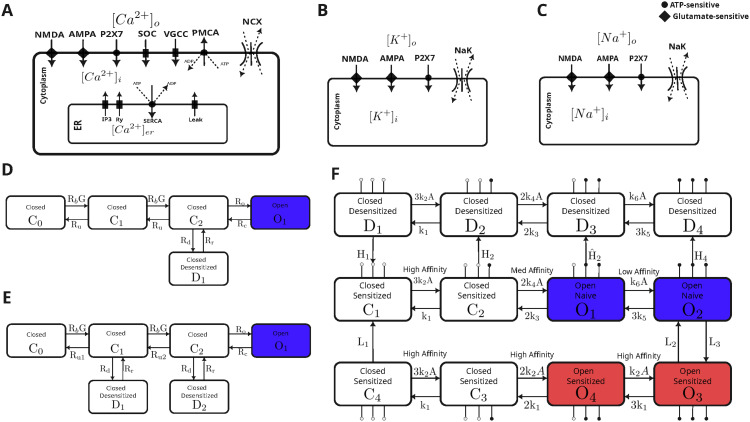
Schematic of the SMM and receptor-specific kinetic Markov models integrated within the SSM. **(A)** Diagram of the cell along with the Ca^2+^ fluxes across the plasma and ER membranes. **(B)** Diagram of the cell along with the K^+^ fluxes across the plasma membrane. **(C)** Diagram of the cell along with the Na^+^ fluxes across the plasma membrane. **(D)** Kinetics of the NMDAR. **(E)** Kinetics of the AMPAR. **(F)** Kinetics of the P2X7R. The kinetics of the three receptors in D, E and F are described in terms of the states $C_{i}$ representing the non-conducting closed states (*i* = 0,1,2 for NMDAR and AMPAR, and *i* = 1,2,3,4 for P2X7R), $D_{i}$ representing the non-conducting desensitized states (*i* = 1 for NMDAR, *i* = 1,2 for AMPAR and *i* = 1,2,3,4 for P2X7R) and $O_{i}$ representing the conducting open (blue) and sensitized (red) states (*i* = 1 for NMDAR and AMPAR, and *i* = 1,2,3,4 for P2X7R), with transitions rates: $R_{b},R_{u},R_{0},R_{c},R_{d},R_{r}$ for NMDAR, $R_{b},R_{u},R_{0},R_{c},R_{d},R_{r}$ for AMPAR and $k_{j},H_{m},L_{n}$ (j=1,…6, *m* = 1,2 and *n* = 1,2,3) for P2X7R, where *G*= [Glut] and *A*= [ATP] are the concentrations of glutamate and ATP, respectively.

A stochastic spatiotemporal model (SSM) describing the dynamics of intrinsic (or voltage-independent) SCaLTs in OPCs in one spatial dimension was previously developed [12]. We extended this model in the current study to account for evoked Ca^2+^ responses, which also entails considering the concentrations of various cations inside and outside the OPCs, including Ca^2+^, Na^+^ and potassium (K^+^) (Fig 1A-1C). Two sets of fluxes were incorporated into the model (Fig 1A): (i) Those occurring at the cell membrane, through voltage gated Ca^2+^ channels (VGCC) including L-type and T-type Ca^2+^ channels ($J_{L-type}$ and $J_{T-type}$, respectively), store operated channels ($J_{SOCE}$), glutamatergic AMPARs and NMDARs ($J_{AMPA}$ and $J_{NMDA}$, respectively), purinergic P2X7Rs ($J_{P2X7}$), Na^+^/Ca^2+^ exchangers ($J_{NCX}$), Na^+^/K^+^ exchangers ($J_{NaK}$), leak across the pasma membrane (*J_Leak,PM_*), Na^+^ leak ($J_{NaLeak}$), inward rectifying K^+^ channels, and PMCA pumps ($J_{PMCA}$). (ii) Those occurring at the ER membrane, through IP3Rs ($J_{IP3}$), RyRs ($J_{Ry}$), Ca^2+^ leak ($J_{Leak}$) and SERCA pumps ($J_{SERCA}$).

The resulting extended model was formulated using a set of stochastic ordinary and partial differential equations that capture the dynamics of cytosolic Ca^2+^ ([Ca^2+^]_*i*_), ER Ca^2+^ ([Ca^2+^]_*ER*_), as well as cytosolic Na^+^ ([Na^+^]_*i*_) and K^+^ ([K^+^]_*i*_) concentrations. Diffusion was explicitly incorporated into the cytosolic Ca^2+^ equation as described below


$$\begin{array}{c} \begin{array}{cc} \frac{\partial [{Ca}^{2+}{]}_{i}}{\partial t}=\;\;\;\; & \rho \frac{\partial^{2}[{Ca}^{2+}{]}_{i}}{\partial x^{2}}+f_{i}(J_{IP3}+J_{Leak}+J_{Leak,PM}+J_{Ry}+J_{NCX}\\ \;\; & -\;J_{PMCA}-J_{SERCA}+J_{P2X7,Ca}+J_{SOCE}+J_{NMDA,Ca}\\ \;\; & +\;J_{AMPA,Ca}+J_{L-type}+J_{T-type}) \end{array} \end{array}$$ (1)

$$\begin{array}{c} \frac{d[{Ca}^{2+}{]}_{ER}}{dt}=-\gamma f_{e}\left(J_{IP3}+J_{Leak}+J_{Ry}-J_{SERCA}\right) \end{array}$$ (2)

$$\begin{array}{c} \begin{array}{cc} \frac{\partial [{Na}^{+}{]}_{i}}{\partial t}=\;\;\;\; & -3J_{NCX}-3J_{NaK}+J_{P2X7,Na}+J_{R,Na}+J_{AMPA,Na}\\ \;\; & +\;J_{NaLeak}+J_{NMDA,Na} \end{array} \end{array}$$ (3)

$$\begin{array}{c} \begin{array}{cc} \frac{\partial [K^{+}{]}_{i}}{\partial t}=\;\;\;\; & 2J_{NaK}-J_{P2X7,K}+J_{R,K}+J_{K}-J_{AMPA,K}\\ \;\; & -\;J_{NMDA,K,} \end{array} \end{array}$$ (4)

where $J_{P2X7,X},J_{NMDA,X},J_{AMPA,X}$ are the contributions of P2X7R, NMDAR and AMPAR, respectively, to the influx of Ca^2+^ (*X* = *Ca*) and Na^+^ (*X* = *Na*), and efflux of K^+^ (*X* = *K*), whereas ρ is the effective diffusion coefficient of free Ca^2+^ in the buffered cytosol rather than the aqueous free-ion diffusion coefficient [37] (assumed to be roughly 50 $\mu {\text{m}}^{2}$/s [38]). The spatial domain for $[{\text{Ca}}^{2+}{]}_{\text{i}}$ extends along a one-dimensional cell of length *L* = 8 μm, subject to no-flux (Neumann) boundary conditions.

The Li-Rinzel model for IP3R kinetics [39] was used to describe $J_{IP3}$ that depends on both [Ca^2+^]_*i*_ and cytosolic IP3 concentration [IP3]_*i*_. Specifically,


$$\begin{array}{c} J_{IP3}=v_{IP3}m_{\infty }^{3}n_{\infty }^{3}h^{3}\left([{Ca}^{2+}{]}_{\mathrm{ER}}-[{Ca}^{2+}{]}_{i}\right), \end{array}$$ (5)

where


$$\begin{array}{c} m_{\infty }=\frac{[IP3{]}_{i}}{[IP3{]}_{i}+d_{1}}\;n_{\infty }=\frac{[{Ca}^{2+}{]}_{i}}{[{Ca}^{2+}{]}_{i}+d_{5}} \end{array}$$

and the gating variable *h* satisfies


$$\begin{array}{c} \frac{dh}{dt}=\frac{h_{\infty }-h}{\tau_{h}}+|\;\eta_{u}\;| \end{array}$$ (6)

with


$$\begin{array}{c} h_{\infty }=\frac{Q_{2}}{Q_{2}+[{Ca}^{2+}{]}_{i}} \end{array}$$

$$\begin{array}{c} \tau_{h}=\frac{1}{a\left(Q_{2}+[{Ca}^{2+}{]}_{i}\right)} \end{array}$$

$$\begin{array}{c} Q_{2}=d_{2}\frac{[IP3{]}_{i}+d_{1}}{[IP3{]}_{i}+d_{3}} \end{array}$$

and [IP3]_*i*_ satisfies the equation


$$\begin{array}{c} \frac{d[IP3{]}_{i}}{dt}=\alpha_{ATP}\frac{\left[ATP\right]}{[ATP]+k_{ATP}}+\alpha_{Glut}\frac{\left[Glut\right]}{[Glut]+k_{Glut}}-\gamma_{IP3}[IP3{]}_{i}, \end{array}$$

where [ATP] and [Glut] are the concentrations of ATP and glutamate applied. Note that [IP3]_*i*_ should typically range between 0.04 to 1.5 μM under physiological conditions [39–41].

The term $\eta_{u}$ in Eq. (6) is an Ornstein-Uhlenbeck noise process that satisfies the equation


$$\begin{array}{c} \frac{d\eta_{u}}{dt}=(-\eta_{u}/\tau_{c})+\sqrt{2D/\tau_{c}}W_{t}, \end{array}$$ (7)

where $\tau_{c}$ is the characteristic correlation time of the noise, *D* is the noise intensity and $W_{t}$ is a Gaussian white noise process with μ=0 and σ=1 [12]. This noise was used to produce the slow baseline oscillations in Ca^2+^ due to the stochastic slow clustering of IP3Rs [12]. Our choice to model noise through the IP3R inactivation dynamics is based on previous studies [12,42]. Specifically, we used the subunit noise in the form of Ornstein-Uhlenbeck noise process to account for the fast opening and closing of the channel and the slow clustering of IP3Rs.

The flux through RyRs ($J_{Ry}$) was described according to the Levine-Keizer model [43], given by


$$\begin{array}{c} J_{Ry}=v_{r}w\left(1+{\left(\frac{[{\text{Ca}}^{2+}{]}_{\text{i}}}{k_{b}}\right)}^{3}\right)/\left(1+{\left(\frac{k_{a}}{[{\text{Ca}}^{2+}{]}_{\text{i}}}\right)}^{4}+{\left(\frac{[{\text{Ca}}^{2+}{]}_{\text{i}}}{k_{b}}\right)}^{3}\right)\left([{\text{Ca}}^{2+}{]}_{\text{ER}}-[{\text{Ca}}^{2+}{]}_{\text{i}}\right) \end{array}$$ (8)

where *w* is a slow gating variable that satisfies


$$\begin{array}{c} \frac{dw}{dt}=\frac{w_{\infty }-w}{\tau_{w}} \end{array}$$ (9)

with


$$\begin{array}{c} w_{\infty }=\frac{\left(1+(k_{a}/[{\text{Ca}}^{2+}{]}_{\text{i}}{)}^{4}+([{\text{Ca}}^{2+}{]}_{\text{i}}/k_{b}{)}^{3}\right)}{1+1/k_{c}+((k_{a}/[{\text{Ca}}^{2+}{]}_{\text{i}}{)}^{4}+([{\text{Ca}}^{2+}{]}_{\text{i}}^{3}/k_{b}{)}^{3})} \end{array}$$

and


$$\begin{array}{c} \tau_{w}=\frac{w_{\infty }}{k_{c}^{-}}. \end{array}$$

A Hill function was used to describe the fluxes through the PMCA and SERCA pumps [44], according to the equations


$$\begin{array}{c} J_{\phi }=v_{\phi }\frac{[{\text{Ca}}^{2+}{]}_{\text{i}}^{2}}{k_{\phi }^{2}+[{\text{Ca}}^{2+}{]}_{\text{i}}^{2}},\;\phi =PMCA,SERCA, \end{array}$$ (10)

whereas the leak Ca^2+^ flux between the cytosol and ER and the external leak into the cytosol across the plasma membrane are described as


$$\begin{array}{c} J_{Leak}=v_{Leak}([{Ca}^{2+}{]}_{\mathrm{ER}}-[{Ca}^{2+}{]}_{i}) \end{array}$$ (11)

$$\begin{array}{c} J_{Leak,PM}=v_{Leak,PM}([{Ca}^{2+}{]}_{o}-[{Ca}^{2+}{]}_{i}), \end{array}$$ (12)

where $[{Ca}^{2+}{]}_{o}$ is the extracellular Ca^2+^ concentration.

The two concentration variables $[{\text{Na}}^{+}{]}_{i}$ and and $[{\text{K}}^{+}{]}_{i}$ were assumed to return to rest according to the equations


$$\begin{array}{c} J_{R,X}=\frac{X_{R}-[X{]}_{i}}{\theta_{X}},\;X={Na}^{+},K^{+}. \end{array}$$ (13)

Here ${\text{Na}}_{R}^{+}$ and ${\text{K}}_{R}^{+}$ represent the resting concentrations of Na^+^ and K^+^, respectively, while $\theta_{Na}$ and $\theta_{K}$ are the time constants of the concentrations of these two ions to return to rest.

To define the remaining fluxes in Eqs. (1)-(4), we first need to introduce the equation for membrane voltage using the Hodgkin-Huxley formalism [45], given by


$$\begin{array}{c} \begin{array}{ccc} C_{m}\;S_{f}\frac{dV}{dt} & = & -(I_{AMPA}+I_{NMDA}+I_{P2X7}+I_{K}+I_{Leak}+I_{SOCE}+I_{L-type}+I_{T-type}\\ & & \;\;+I_{NaK}+I_{NCX}+I_{NaLeak}) \end{array} \end{array}$$ (14)

where $I_{Y}$ (Y=AMPA,NMDA,P2X7,K,Leak,SOCE,L−type,T−type,NaK,NCX and *NaLeak*) are the various ionic currents produced by ion flow through channels and exchangers expressed on the membrane of OPCs, $S_{f}$ is the membrane surface and $C_{m}$ is the specific membrane capacitance. Consistent with previous electrophysiological studies of oligodendrocyte-lineage glial cells [46,47] that demonstrated predominantly passive, non-regenerative membrane behaviour and negligible internal voltage gradients, we treated the membrane potential in this study as spatially uniform in our Hodgkin–Huxley formalism, incorporating the relevant ionic currents. The fluxes associated with these currents (including those for the two VGCCs: $I_{L-type}$ and $I_{T-type}$) were computed using the equation [44]


$$\begin{array}{c} J_{Y}=\frac{1}{z\overset{―}{V}F}I_{Y}, \end{array}$$ (15)

where *z* is the valence (*z* = 1 for K^+^ and Na^+^, and *z* = 2 for Ca^2+^), $\overset{―}{V}$ is the cell volume, and *F* is Faraday’s constant.

We used Destexhe et al. NMDAR and AMPAR models [48] (Fig 1D and 1E, respectively) to describe $I_{NMDA}$ and $I_{AMPA}$, given by


$$\begin{array}{c} I_{NMDA}={\overset{―}{g}}_{NMDA}OB(V)(V-E_{NMDA}) \end{array}$$ (16)

$$\begin{array}{c} I_{AMPA}={\overset{―}{g}}_{AMPA}O(V-E_{AMPA}), \end{array}$$ (17)

where *O* is the open state of each receptor, $E_{Y}$ and ${\overset{―}{g}}_{Y}$ are, respectively, the Nernst potential and maximum conductance of NMDARs (*Y* = *NMDA*) and AMPARs (*Y* = *AMPA*), and *B*(*V*) is the voltage-dependent magnesium (Mg^2+^) block, given by


$$\begin{array}{c} B(V)=\frac{1}{1+\exp(-0.062V)[Mg^{2+}{]}_{o}/3.57}. \end{array}$$

where [Mg^2+^]_*o*_ is the extracellular Mg^2+^ concentration. Note that AMPAR and NMDAR internalization is ignored, because they both occur at a much slower timescale (minutes to tens of minutes) compared to the second-timescale of Ca^2+^ transients [49,50]. Furthermore, their Nernst potentials are set to zero because these two receptors are nonspecific [48].

Using the Khadra et al. P2X7R model [29] to describe $I_{P2X7}$ (Fig 1F), we have


$$\begin{array}{c} I_{P2X7}={\overset{―}{g}}_{P2X7}(O_{1}+O_{2}+O_{3}+O_{4})(V-E_{P2X7}), \end{array}$$ (18)

where $O_{1},O_{2}$ are the open states and $O_{3},O_{4}$ are the sensitized states of P2X7R, ${\overset{―}{g}}_{P2X7}$ is the receptor maximum conductance and $E_{P2X7}$ is its Nernst potential (set to zero because P2X7Rs are nonspecific [28]).

For the inward-rectifying K^+^ current ($I_{K}$), we adopted the formalism of [51] to describe it, i.e.,


$$\begin{array}{c} I_{K}={\overset{―}{g}}_{K}\sqrt{[K^{+}{]}_{o}}(V-E_{K}), \end{array}$$ (19)

where $[K^{+}{]}_{o}$ is the extracellular K^+^ concentration, ${\overset{―}{g}}_{K}$ is the maximum conductance of $I_{K}$, and $E_{K}$ is the K^+^ Nernst potential that depends on both the gas constant (*R*) and absolute temperature ($\overset{―}{T}$) as follows


$$\begin{array}{c} E_{K}=\frac{R\overset{―}{T}}{F}\log\frac{[K^{+}{]}_{o}}{[K^{+}{]}_{i}}. \end{array}$$

**Fig 2:**
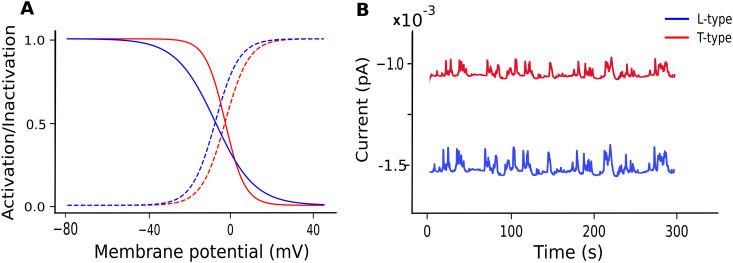
Kinetics of the L-type and T-type Ca^2^^+^ channels. **(A)** The steady state activation and inactivation curves of the L-type and T-type Ca^2+^ channels color-coded according to the legend. **(B)** The stochastic current fluctuations produced by the L-type and T-type Ca^2+^ channels color-coded according to the legend.

Similarly, we adopted the formalisms from [52] and reparameterized them using the data from [20] to describe the two VGCCs: L-type and T-type Ca^2+^ currents ($I_{L\text{-}type}$ and $I_{T\text{-}type}$, respectively, included in the SSM Fig 2).

In this case,


$$\begin{array}{c} I_{L-type}={\overset{―}{g}}_{L}m_{L}h_{L}(V-E_{Ca}) \end{array}$$ (20)

$$\begin{array}{c} I_{T-type}={\overset{―}{g}}_{T}m_{T}h_{Tf}(V-E_{Ca}), \end{array}$$ (21)

where $E_{ca}$ is Ca^2+^ Nernst potential, given by


$$\begin{array}{c} E_{Ca}=\frac{R\overset{―}{T}}{2F}\log\frac{[{Ca}^{2+}{]}_{o}}{[{Ca}^{2+}{]}_{i}}, \end{array}$$

and ${\overset{―}{g}}_{\zeta }$ is the maximum conductance of $I_{L}$ (ζ=L) and $I_{T}$ (ζ=T). The two gating variables $m_{L}$ and $h_{L}$ are the activation and inactivation variables of $I_{L}$, respectively; their dynamics are governed by the equations


$$\begin{array}{c} \frac{dX}{dt}=\frac{\overset{―}{X}-X}{\tau_{X}}, \end{array}$$ (22)

where $\overset{―}{X}={\overset{―}{m}}_{L},{\overset{―}{h}}_{L}$ are the steady state of activation and inactivation, respectively, and $\tau_{X}=\tau_{m_{L}},\tau_{h_{L}}$ are the time constants, given by


$$\begin{array}{c} \begin{array}{ccc} {\overset{―}{m}}_{L} & = & 1/(1+\exp(-V/6))\\ {\overset{―}{h}}_{L} & = & 1/(1+\exp(V/5))\\ \tau_{m_{L}} & = & 18\exp(-((V+45)/20{)}^{2}+10)/10\\ \tau_{h_{L}} & = & 10\exp(-((V+100)/10{)}^{2})+20)/10. \end{array} \end{array}$$

The gating variables of the current $I_{T}$, on the other hand, are the activation variable $m_{T}$ and the inactivation variable $h_{Tf}$; their dynamics are governed by Eq. (22), where $\overset{―}{X}={\overset{―}{m}}_{T},{\overset{―}{h}}_{Tf}$ are the steady state activation and inactivation, respectively, and $\tau_{X}=\tau_{m_{T}},\tau_{h_{Tf}}$ are the time constants, given by


$$\begin{array}{ccc} {\overset{―}{m}}_{T} & = & 1/(1+\exp(-(V+5)/6))\\ {\overset{―}{h}}_{Tf} & = & 1/(1+\exp((V+5)/10))\\ \tau_{m_{T}} & = & \exp(-((V+5)/6{)}^{2})+2\\ \tau_{h_{Tf}} & = & \exp(-((V+5)/10{)}^{2})+2. \end{array}$$

The SOCE current ($I_{SOCE}$) expressed in [53] was adopted in this study; its expression is given by


$$\begin{array}{c} I_{SOCE}={\overset{―}{g}}_{SOCE}\frac{\tanh([{Ca}^{2+}{]}_{er}-k_{SOCE})}{2}(V-E_{Ca}). \end{array}$$ (23)

Two leak currents were included in the voltage equation (14): a Na^+^ leak current ($I_{NaLeak}$) and a standard leak current ($I_{Leak}$). These two currents are given by


$$\begin{array}{c} I_{NaLeak}={\overset{―}{g}}_{NaLeak}(V-E_{NaLeak}) \end{array}$$ (24)

$$\begin{array}{c} I_{Leak}={\overset{―}{g}}_{L}(V-E_{Leak}), \end{array}$$ (25)

where $E_{NaLeak},E_{Leak}$ are Nernst potential of these currents, whereas $g_{NaLeak}$ and $g_{Leak}$ are their conductances.

Finally, two exchangers were included in the model, the ${\text{Na}}^{+}/{\text{Ca}}^{2+}$ exchanger ($I_{NCX}$) and the ${\text{K}}^{+}/{\text{Na}}^{+}$ exchanger ($I_{NaK}$). Using the formalisms for these two currents introduced in [54] and making them voltage dependent, we have:


$$\begin{array}{c} I_{NCX}=\frac{{\overset{―}{g}}_{NCX}}{1+{\left(\frac{K_{NCX,Ca}}{[{Ca}^{2+}{]}_{i}}\right)}^{1.5}}\frac{\left([{Na}^{+}{]}_{i}^{3}[{Ca}^{2+}{]}_{o}\exp(\frac{\eta zVF}{RT})-[{Na}^{+}{]}_{o}^{3}[{Ca}^{2+}{]}_{i}\exp(\frac{(\eta -1)VzF}{RT})\right)}{\left(1+d_{NCX}([{Na}^{+}{]}_{o}^{3}[{Ca}^{2+}{]}_{i}+[{Na}^{+}{]}_{i}^{3}[{Ca}^{2+}{]}_{o})\right)} \end{array}$$ (26)

$$\begin{array}{c} I_{NaK}={\overset{―}{I}}_{NaK}\frac{[K^{+}{]}_{o}}{\left([K^{+}{]}_{o}+K_{NaK,Ko}\right)}\frac{[{Na}^{+}{]}_{i}^{1.5}}{[{Na}^{+}{]}_{i}^{1.5}+K_{Nak,Nai}^{1.5}}\frac{V+135.1}{V+300}, \end{array}$$ (27)

where [Na^+^]_*o*_ is the extracellular Na^+^ concentration, η is the position of the energy barrier, ${\overset{―}{I}}_{NCX}$ is the maximum current of ${\text{Na}}^{+}/{\text{Ca}}^{2+}$ exchanger, ${\overset{―}{I}}_{NaK}$ is the maximum current of ${\text{K}}^{+}/{\text{Na}}^{+}$ exchanger, and $K_{NCX,Ca},d_{NCX},K_{NaK,Ko}$ and $K_{NaK,Nai}$ are parameters representing half-maximum activation. The receptors are uniformly distributed along the membrane (spatial axis). Unless otherwise stated, all time series presented in this paper were obtained from the mid point of the cell (at *x* = 4) for a cell of length 8.

Parameter values of this stochastic spatiotemporal model (SSM), given by Eqs. (1)-(27), and their definitions are provided in Tables 1 and 2. By ignoring noise, i.e., removing Eq. (7), and by setting diffusion to zero (i.e., ρ=0), we obtain the deterministic temporal model (DTM). In the absence of membrane voltage dynamics and the kinetics of P2X7Rs, AMPARs and NMDARs, the SSM was able to reproduce the original features of the model associated with intrinsic ScaLTs previously reported [12] (S1 Fig).

**Table 1:** Model parameters.

Parameter	Value	Unit
**Cytosolic Ca**^**2 +**^ **dynamics** (*d*[*Ca*^2+^]/*dt*)		
ρ	0.00005	mm^2^/s
$f_{i}$	0.05	–
**ER Ca**^**2 +**^ **dynamics** $\left(d[Ca^{2+}{]}_{ER}/dt\right)$		
γ	9	–
$f_{e}$	0.05	–
**IP**_**3**_ **receptor flux** $\left(J_{IP3}\right)$		
$v_{IP3}$	0.88	s^−1^
*d*_1_	0.13	μM
*d*_5_	0.08234	μM
*a*	0.2	s/μM
*d*_3_	0.9434	μM
*d*_2_	1.049	μM
$\alpha_{ATP}$	0.03	μM/s
$k_{ATP}$	1	mM
$\alpha_{glut}$	0.3	μM/s
$k_{glut}$	1	mM
$\gamma_{IP3}$	0.01	s^−1^
$\tau_{c}$	0.01	s
*D*	0.4	–
**ER leak flux** $\left(J_{leak}\right)$		
$v_{Leak}$	0.5	s^−1^
**Ryanodine receptor flux** $\left(J_{RyR}\right)$		
$v_{r}$	18	s^−1^
$k_{b}$	0.2573	μM
$k_{a}$	0.0192	μM
$k_{c}$	0.0571	–
${\bar{k}}_{c}$	0.0571	s
**PMCA and SERCA pumps**		
*v*_3_	120	s^−1^
$h_{c3}$	2	–
*k*_3,*serc*_	0.3	μM
$v_{pmca}$	0.6	s^−1^
$k_{m,pmca}$	0.8	μM
**Return currents**		
$\theta_{Na}$	10	s
$Na_{R}$	12	mM
$\theta_{K}$	10	s
$K_{R}$	120	mM

**Table 2:** Model parameters for HH formalism, receptors, channels, and transporters.

Parameter	Value	Unit	Parameter	Value	Unit
**HH equation**			**Potassium current** ($I_{K}$)		
$C_{m}$	1	μF·cm^−2^	${\bar{g}}_{K}$	11.58	nS
$S_{f}$	5000	$\mu {\text{m}}^{2}$	$[K^{+}{]}_{o}$	4	mM
*z*	1, 2	–	*R*	8.314	J·K^−1^·mol^−1^
$\bar{V}$	6.5	pL	*T*	300	K
*F*	96485.3321	C·mol^−1^			
**NMDA receptor**			**Voltage-gated Ca**^**2 +**^ **channels**		
${\bar{g}}_{NMDA}$	1.4	nS	$g_{T}$	0.5	nS
$E_{NMDA}$	0	mV	$g_{L}$	0.8	nS
$R_{b}$	8333.33	${\text{s}}^{-1}\cdot {\text{M}}^{-1}$	**SOCE**		
*R*_0_	46.5	s^−1^	$g_{SOCE}$	1	nS
$R_{u}$	0.125	s^−1^	$k_{SOCE}$	0.939	μM
$R_{c}$	7.38	s^−1^			
$R_{d}$	0.014	s^−1^	**Na,Ca leak** ($I_{K,\text{leak}}$)		
$R_{r}$	0.1133	s^−1^	$E_{NaLeak}$	60	mV
$[Mg{]}_{o}$	1	mM	$g_{leakK}$	2	nS
**AMPA receptor**			**Leak** (*I*_leak_)		
${\bar{g}}_{AMPA}$	3	nS	$v_{leak}$	1	nS
$E_{AMPA}$	0	mV	$E_{leak}$	-81	mV
$R_{b}$	10000	${\text{s}}^{-1}\cdot {\text{M}}^{-1}$	$v_{leak,PM}$	0.016	pS
*R*_0_	27	s^−1^	**Na**^**+**^**/Ca**^**2+**^ **exchanger** ($I_{NCX}$)		
$R_{u1}$	0.0766	s^−1^	$d_{NCX}$	0.25	–
$R_{u2}$	114.67	s^−1^	${\bar{g}}_{NCX}$	0.04	nS
$R_{c}$	0.4	s^−1^	$K_{NCX,Ca}$	1.5	*m*M
$R_{d}$	0.09	s^−1^	$[Na^{+}{]}_{o}$	140	*m*M
$R_{r}$	0.0128	s^−1^	$[Ca^{2+}{]}_{o}$	2.5	*m*M
**P2X7 receptor**			**Na^+^/K^+^ pump** ($I_{NaK}$)		
${\bar{g}}_{P2X7}$	22.5	nS	${\bar{I}}_{NaK}$	22.6	pA
$E_{P2X7}$	0	mV	$K_{NaK,K_{o}}$	1.32	mM
*k*_1_	0.3	s^−1^	$K_{NaK,Na}$	14.5	mM
*k*_2_	1260	${\text{s}}^{-1}\cdot {\text{M}}^{-1}$			
*k*_3_	2.4	s^−1^			
*k*_4_	1575	${\text{s}}^{-1}\cdot {\text{M}}^{-1}$			
*k*_5_	1.58	s^−1^			
*k*_6_	221	${\text{s}}^{-1}\cdot {\text{M}}^{-1}$			
*L*_1_	0.0001	s^−1^			
*L*_2_	0.004	s^−1^			
*L*_3_	0.05	s^−1^			
*H*_1_	0.001	s^−1^			
*H*_2_	0.01	s^−1^			
${\hat{H}}_{2}$	0.1	s^−1^			
*H*_4_	0.6	s^−1^			

#### 2.2.2 Random ATP and glutamate stimulations.

The SSM model Eqs. (1)-(27) was subjected to random ATP and glutamate stimulations uniformly across the entire length of the cell (the one-dimensional spatial domain of length *L*). These random stimulations were modeled as square-wave pulses in the form of spike trains with varying amplitudes and frequencies (see representative trances in S2 Fig). Event occurrence was modeled using Bernoulli trials (i.e., a binomial distribution ℬ(n,p) with *n* = 1) evaluated every 0.01 s (the simulation time step) of simulation time, with probabilities *p* = 0.001, 0.0025, and 0.005 corresponding to low, medium, and high event rates (with expected interspike intervals of approximately 10, 4, and 2 s, respectively). At each time step, a sampled value of 1 triggers the onset of a stimulation event, whereas a sampled value of 0 results in no stimulation, and the process is repeated at the next time step. Each stimulation event, once initiated, lasts for 0.1 s. The amplitudes of individual events of ATP and glutamate within the spike trains were sampled from a normal distribution $𝒩(0,1/\sqrt{0.5\times 10^{-3}})$ for ATP and $𝒩(0,1/\sqrt{1\times 10^{-3}})$ for glutamate, resulting in an overall input distribution of Bernoulli$\left(p)\times 𝒩(0,\sqrt{0.5\times 10^{-3}}\right)$ or Bernoulli$\left(p)\times 𝒩(0,\sqrt{1\times 10^{-3}}\right)$, respectively. To analyze the simulated Ca^2+^ signals under each stimulation condition, we conducted 50 simulations, each lasting 300 s, and recorded Ca^2+^ transients exceeding 0.11 μM. The threshold was established based on the mean Ca^2+^ concentration in wild-type simulations, corresponding to the default parameter values in Tables 1 and 2. The amplitudes of collected Ca^2+^ transients were then binned to construct a distribution. Similarly, the width of these transients were quantified by computing their full width at half maximum (FWHM) and the results were binned to construct a distribution.

#### 2.2.3 Numerical methods and software.

A forward time-centered space scheme with Neumann boundary conditions was used to compute numerical solutions to the SSM. A custom-made python script was used to compute these solutions. The DTM was numerically solved in XPPAUT (a freeware available online at http://www.math.pitt.edu/bard/xpp/xpp.html). The timeseries of all simulated Ca^2+^ signals were extracted from a single spatial point at the midpoint of the domain *L* (i.e., at *x* = 4), representing the one-dimensional cell. Ca^2+^ transients in these simulated time series were classified as Ca^2+^ spikes if they exceeded a threshold, defined as the mean of the simulated signal generated by the SSM under default parameter values (also referred to as the WT model). To compare spikes across different conditions, such as the WT model versus perturbed versions where L- and T-type Ca^2+^ channels are blocked (a version that is referred to as the KO model), the average threshold from WT simulations was used as a reference for both conditions. To perform clustering analysis, each time series was fitted with a 4th-degree polynomial, and the resulting polynomial coefficients were used as input for a k-means algorithm. The optimal cluster number was determined to be three using the elbow score.

### 2.3 Quantification of Ca^2+^ Transients

SCaLTs (including those facilitated by the voltage) were isolated by eliminating Ca^2+^ fluxes associated with P2X7Rs, AMPARs and NMDARs from the SSM, as these fluxes are responsible for evoked responses. Following the approach in [12], Ca^2+^ transients, including SCaLTs and those influenced by fluxes involved in evoked responses, were identified by detecting peaks exceeding 20% above baseline or 1 standard deviation above the mean. To enable direct comparison between simulated and experimental data, the time series of both datasets (*S*(*t*)) were normalized between 0 and 1 using the equation.


$$\begin{array}{c} S_{norm}=\frac{S(t)-\min(S(t))}{\max(S(t))-\min(S(t))}, \end{array}$$

## 3 Results

### 3.1 Bifurcation analysis of evoked responses

We extended our previously developed stochastic spatiotemporal flux-balance model (SSM) describing Ca^2+^ dynamics in OPCs along a one-dimensional domain [12] to include both membrane voltage dynamics and the effects of ATP and glutamate on evoked Ca^2+^ transients. To analyze the underlying deterministic mechanisms, we derived a reduced deterministic temporal model (DTM) by removing spatial diffusion and stochastic noise from the SSM.

Incorporating the effects of ATP and glutamate concentrations ([ATP] and [Glut], respectively) into the model allowed us to investigate how these exogenous stimuli (i.e., neurotransmitters), known to trigger evoked Ca^2+^ responses in OPCs, modulate the deterministic dynamics of the DTM. To this end, we performed bifurcation analyses of the membrane voltage (*V*) and cytosolic Ca^2+^ concentration ($[{\text{Ca}}^{2+}{]}_{\text{i}}$) with respect to both [ATP] and [Glut], using the continuation method in XPPAUT.

**Fig 3:**
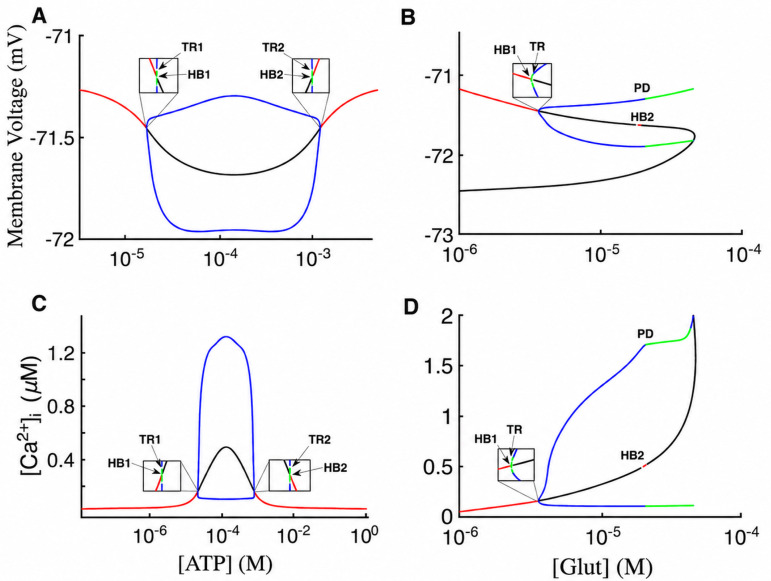
Steady state analysis of the DTM. Bifurcation diagrams of the membrane voltage (*V*) with respect to **(A)** ATP concentration: [ATP], and **(B)** glutamate concentration: [Glut]. Bifurcation diagrams of cytosolic Ca^2+^ concentration ($[{\text{Ca}}^{2+}{]}_{\text{i}}$) with respect to **(C)** [ATP] and **(D)** [Glut]. Red (black) solid lines: Branches of stable (unstable) equilibria. Green (blue) solid lines: Envelopes of stable (unstable) limit cycles; the envelopes in A and C are delimited by two Hopf Bifurcations (HB1 to the left and HB2 to the right) and undergo two torus bifurcations (TR1 to the left and TR2 to the right), while the envelopes in B and D emerge from a Hopf bifucation (HB) and undergo both a torus bifurcation (TR) and a period doubling bifurcation linking the stable and unstable envelopes.

In the case of [ATP], we found that both *V* (Fig 3A) and $[{\text{Ca}}^{2+}{]}_{\text{i}}$ (Fig 3C) exhibit three distinct regimes of behavior at low, intermediate and high [ATP]. At low and high [ATP], a stable branch of equilibria was observed, representing the quiescent state of the cell; these stable branches connect with each other through an unstable branch of equilibria for intermediate [ATP] at two Hopf bifurcations (HB1 to the left and HB2 to the right). Envelopes of stable limit cycles emerge from these two Hopf bifurcations and soon after become envelopes of unstable limit cycles when they undergo torus bifurcations (TR1 to the left and TR2 to the right), signifying the onset of chaotic dynamics. This latter regime corresponds to where ATP-evoked periodic responses in cytosolic Ca^2+^ can be observed in the form of irregularly-shaped pikes (S3 Fig). It is important to note that the quiescent regime to the left of HB1 is type III excitable allowing the model to generate SCaLTs reminiscent to those previously analyzed in [12].

By repeating the same analysis with [Glut], we also obtained three regimes of behavior for both *V* (Fig 3B) and $[{\text{Ca}}^{2+}{]}_{\text{i}}$ (Fig 3D): a quiescent regime at low [GLUT] defined by a stable branch of equilibria possessing type III excitability (allowing the model to produce SCaLTs), a mixed-mode oscillation regime at intermediate [GLUT] (see time series simulations in S4A Fig) defined by envelopes of limit cycles that emerge from a Hopf bifurcation (HB) and immediately undergo torus bifurcation (TR) before terminating at a period-doubling (PD) bifurcation, and a tonically spiking oscillatory regime at high [GLUT] (see time series simulations in S4B Fig) formed by envelopes of stable limit cycles emerging from the PD and terminating at a homoclinic bifurcation. The spikes in this periodic regime get wider closer to the homoclinic (S4C Fig). The stable branch of equilibria at low [GLUT] regime losses stability at the HB and persists at intermediate and high [GLUT], while the periodic orbits in the mixed-mode oscillation regime combine both small and large amplitude oscillations within each cycle in a manner reminiscent to those previously observed [12,55] (S4A Fig).

Together, these results demonstrate that both [ATP] and [Glut] can drive the system into regimes characterized by periodic Ca^2+^ spiking, thereby accounting for evoked cytosolic Ca^2+^ responses in OPCs.

### 3.2 Effects of L- and T-type Ca^2+^ channels on SCaLTs

It has been previously shown, using computational modeling that Ca^2+^-induced Ca^2+^-release (CICR) through IP3R and RyR, along with SOCE and NCX fluxes on the plasma membrane and fluxes through SERCA and PMCA pumps, are sufficient for intrinsically generating SCaLTs in OPCs [12]. The flux balance model accounting for these fluxes successfully reproduced key features of Ca^2+^ signals, including SCaLT characteristics (e.g., slow baseline oscillations, doublets, random spiking, etc). This model, however, did not include the effects of VGCC expressed on OPC plasma membrane, a component that may have significant effects on SCaLTs.

**Fig 4:**
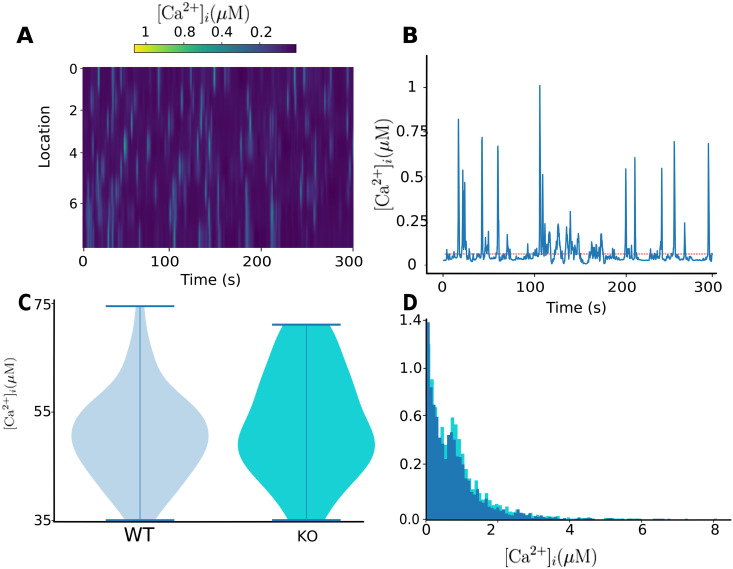
Contribution of L- and T-type Ca^2^^+^ channels to SCaLTs in OPCs as predicted by the SSM. **(A)** Heatmap of the spatiotemporal dynamics of Ca^2+^ concentration along the entire length of the cell *L* = 8 μm, color-coded according to the color scale on top. The transient Ca^2+^ events in the form of short-lived waves illustrate the spontaneous Ca^2+^ local transients (SCaLTs) occurring along the spatial domain during a 300-s simulation. **(B)** A simulated stochastic Ca^2+^ signal generated by the SSM displaying sub- and supra-threshold Ca^2+^ transients separated by a red line marking the mean. The supra-threshold VGCC-mediated Ca^2+^ transients combine the intrinsic and voltage-facilitated SCaLTs. **(C)** Violin plots of the number of SCaLTs generated by the SSM in the presence (labeled WT) and absence (labeled KO) of L- and T-type Ca^2+^ channels. A total of 50 steady state simulations of 60 s each were included per condition and the number of SCaLTs above the average threshold of WT condition were counted for both conditions. Note that SCaLTs in KO condition are essentially the voltage-independent (or intrinsic) SCaLTs. **(D)** Average amplitude distribution of sub- and suprathreshold Ca^2+^ transients for WT SSM (blue) and KO SSM (cyan) obtained from the simulations in **B.**

To investigate this, two types of Ca^2+^ channels were included in the SSM, including the high-voltage activated L-type and low-voltage activated T-type Ca^2+^ channels. Spatiotemporal simulations along the length of the cell *L* revealed short-lived traveling Ca^2+^ waves that propagate briefly before terminating (Fig 4A). Examination of the temporal dynamics of Ca^2+^ at the midpoint of the one-dimensional domain showed persistent random spiking events that surpass a threshold, defined as the mean, reminiscent of SCaLTs (Fig 4B); these spiking events integrate SCaLTs generated intrinsically with those facilitated by the voltage. This is reflected by the small-amplitude fluctuations in Ca^2+^ influx through VGCCs (S5 Fig) that contribute to this facilitation. To further assess the contribution of VGCCs to SCaLTs, we compared the steady-state distribution of spike counts between WT (i.e., with L- and T-type Ca^2+^ channels included in the SSM) and KO (i.e., SSM lacking L- and T-type Ca^2+^ channels) conditions (Fig 4C). Quantification was based on 50 steady-state simulations (60 s each), with the average WT threshold used to identify Ca^2+^ spikes in both conditions. Our results showed that VGCCs increase the number of spikes by expanding the tail of the distribution in WT compared to KO conditions. Closer inspection of the amplitude of all Ca^2+^ transients in these simulations, including sub- and supra-threshold ones, revealed that VGCCs increase the amplitude of spikes by generating a longer tail in WT condition (Fig 4D) but do not affect the subthreshold oscillations. These findings indicate that VGCCs further facilitate SCaLTs and contribute to the generation of more pronounced Ca^2+^ signals.

### 3.3 OPC Ca^2+^ responses Induced by ATP

**Fig 5:**
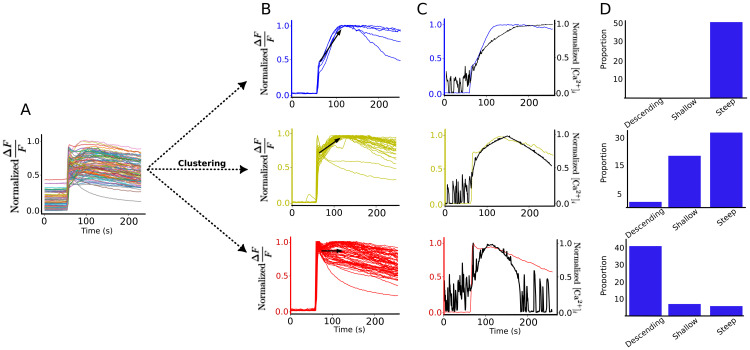
P2X7R-dependent Ca^2^^+^ responses in OPCs upon prolonged ATP stimulation. **(A)** The set of all recorded 99 Ca^2+^ responses - normalized between 0 and 1 - recoded in OPCs bathed in Hanks’ Balanced Salt solution and stimulated with 100 mM ATP without interruption starting at 60 **s. (B)** The three distinct clusters of Ca^2+^ response profiles, including those with steep gradient = 0.009 (blue; top), shallow gradient = 0.04 (yellow; middle) and descending gradient =−0.001 (red; bottom). **(C)** Simulated Ca^2+^ responses (black) - normalized between 0 and 1 - at different ATP concentrations: [ATP]$=0.1\times 10^{-3}$ M (top), $0.055\times 10^{-3}$ M (middle), and $0.043\times 10^{-3}$ M (bottom), overlaid on one representative Ca^2+^ recording from each cluster in **B.** Simulated responses were obtained by applying ATP for 120 s. **(D)** A set of 50 independent simulations of Ca^2+^ responses clustered into steep, shallow and descending gradient groups at different ATP concentrations: [ATP]$=0.1\times 10^{-3}$, (top) $0.055\times 10^{-3}$ (middle) and $0.043\times 10^{-3}$ (bottom). SCaLTs prior to ATP stimulation observed in the simulations in C are absent in the experimental recordings due to the bath solution.

It has been previously shown that OPCs express ATP-gated nonspecific P2X7Rs on their cell membrane [9]. To investigate experimentally how P2X7Rs affect Ca^2+^ responses in these cells, a prolonged step pulse of 100 mM ATP was applied at 60 s, and Ca^2+^ signals from 99 ROIs were recorded. Ca^2+^ transients arising from spontaneous activity were minimized by simultaneously bathing cells in Hanks’ Balanced Salt Solution, known to suppress SCaLTs and generate semi-quiescent Ca^2+^ signals. Despite variability across recordings, the resulting Ca^2+^ responses consistently exhibited a rapid rise characteristic of P2X7R activation (Fig 5A). To better understand the differences among these profiles, we clustered them into three distinct groups (Fig 5B). The main feature distinguishing these clusters was the behavior of the slow component following the fast rise: this component either increased with a relatively steep gradient (top), increased with a shallow gradient (middle), or gradually decreased with a descending gradient (bottom) over time (Fig 5B).

To uncover how P2X7Rs contribute to the distinct response profiles of the three clusters, we performed simulations using the SSM with integrated P2X7R kinetics. Because it is unclear how Hanks’ Balanced Salt solution removes SCaLTs (whether voltage-facilitated or intrinsic) and since P2X7R-mediated evoked Ca^2+^ responses are robust enough to overshadow SCaLTs, we did not modify the model to block these random spiking events. The application of a prolonged step pulse of [ATP]=100 mM (i.e., $0.1\times 10^{-3}$ M) to the model generated responses that matched closely those obtained experimentally with a steep gradient (Fig 5C top, and 5D top). Interestingly, by reducing the amplitude of the ATP step pulse to [ATP]=55 mM and 43 mM, we were able to shift the Ca^2+^ responses produced by the SSM to closely match the experimentally observed profiles of the shallow gradient (Fig 5C middle, and 5D middle) and descending gradient (Fig 5C bottom, and 5D bottom) responses, respectively. Taken together, these results suggest that P2X7Rs mediate the Ca^2+^ signals induced by prolonged ATP stimulation and that not all cells in culture experience the same [ATP] during these stimulation experiments. Moreover, the ability of the lower ATP concentrations to reproduce the spectrum of experimentally observed responses (Fig 5C, middle and bottom; Fig 5D, middle and bottom) suggests that stochasticity also plays a key role in shaping these responses. We emphasize that ATP was applied uniformly across the entire spatial domain representing the full length of the cell model in the SSM.

### 3.4 OPC Ca^2+^ responses induced by glutamate

**Fig 6:**
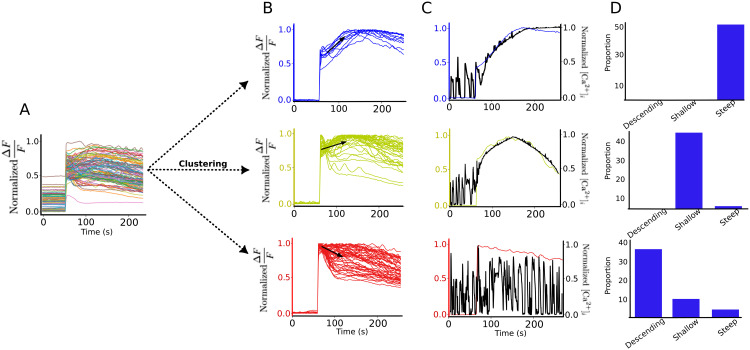
AMPAR and NMDAR-dependent Ca^2^^+^ responses in OPCs upon prolonged glutamate stimulation. **(A)** The set of all 99 recorded Ca^2+^ responses - normalized between 0 and 1 - recoded in OPCs bathed in Hanks’ Balanced Salt solution and stimulated with 100 mM glutamate without interruption starting at 60s. **(B)** The three distinct clusters of Ca^2+^ response profiles, including those with steep gradient = 0.0081 (blue; top), shallow gradient = 0.0015 (yellow; middle) and descending gradient =−0.0047 (red; bottom). **(C)** Simulated Ca^2+^ responses (black) - normalized between 0 and 1 - at different glutamate concentrations: [Glut]$=0.1\times 10^{-3}$ M (top) $0.06\times 10^{-3}$ M (middle), and $0.04\times 10^{-3}$ M (bottom), overlaid on one representative Ca^2+^ recording from each cluster in **B.** Simulated responses were obtained by applying glutamate for 120 **s. (D)** A set of 50 independent simulations of Ca^2+^ responses clustered into steep, shallow and descending gradient groups at different glutamate concentrations: [Glut]=$0.1\times 10^{-3}$, (top) $0.06\times 10^{-3}$ (middle) and $0.04\times 10^{-3}$ (bottom). SCaLTs prior to glutamate stimulation observed in the simulations in C are absent in the experimental recordings due to the bath solution.

As is the case for P2X7Rs, OPCs also express the glutamatergic AMPARs and NMDARs [9]. To determine how glutamate affects Ca^2+^ signals in OPCs, we removed Ca^2+^ transients as before using the Hanks’ Balanced Salt solution and recorded from 99 ROIs in these cells when stimulated with prolonged step pulse of 100 mM glutamate starting at 60 s (Fig 6A). Despite exhibiting the same level of variability as that seen with ATP stimulation, the fast rise in Ca^2+^ responses upon glutamate stimulation was always a consistent feature across all responses. Clustering the recorded signals, as was done before, produced three distinct groups of responses distinguished from each other by the slow component following the fast rise. Once again, the slow component either increased the amplitude of the response with a steep gradient (top), increased it with a shallow gradient (middle) or gradually decreased it with a descending gradient (bottom) over time (Fig 6B).

Simulating the SSM with AMPAR and NMDAR kinetics in response to a prolonged 100 mM ($0.1\times 10^{-3}$ M) glutamate step pulse demonstrated that the model accurately reproduces the experimentally observed steep gradients of Ca^2+^ responses with high fidelity (Fig 6C top and 6D top). This occurs despite the experimental recordings (as with ATP recordings in Fig 5) being performed in Hanks’ Balanced Salt Solution, known to suppress SCaLTs. To capture the shallow gradient responses (Fig 6C middle and 6D middle), we had to reduce [Glut] to 60 mM. Further decreasing [Glut] to 40 mM predominantly produced responses matching the descending gradient profile. Interestingly, some simulations at that lowest glutamate concentration also captured steep and shallow gradient responses. These results thus suggest that the glutamatergic receptors included in the model can reproduce the experimentally observed Ca^2+^ responses and that not all OPCs in culture experience the same glutamate levels. Finally, the findings once again highlight the crucial role of stochasticity in generating the wide spectrum of responses seen experimentally. It is important to emphasize again that glutamate was applied uniformly across the entire spatial domain representing the full length of the cell model in the SSM.

### 3.5 Role of RyR in ATP- and glutamate-dependent Ca^2+^ responses

One important and common feature of the Ca^2+^ responses induced by ATP and glutamate was the presence of an initial fast rise in Ca^2+^ signal upon stimulation. This feature appeared in all clusters of Ca^2+^ responses and in both conditions (Figs 5A, 5B, 6A, and 6B).

**Fig 7:**
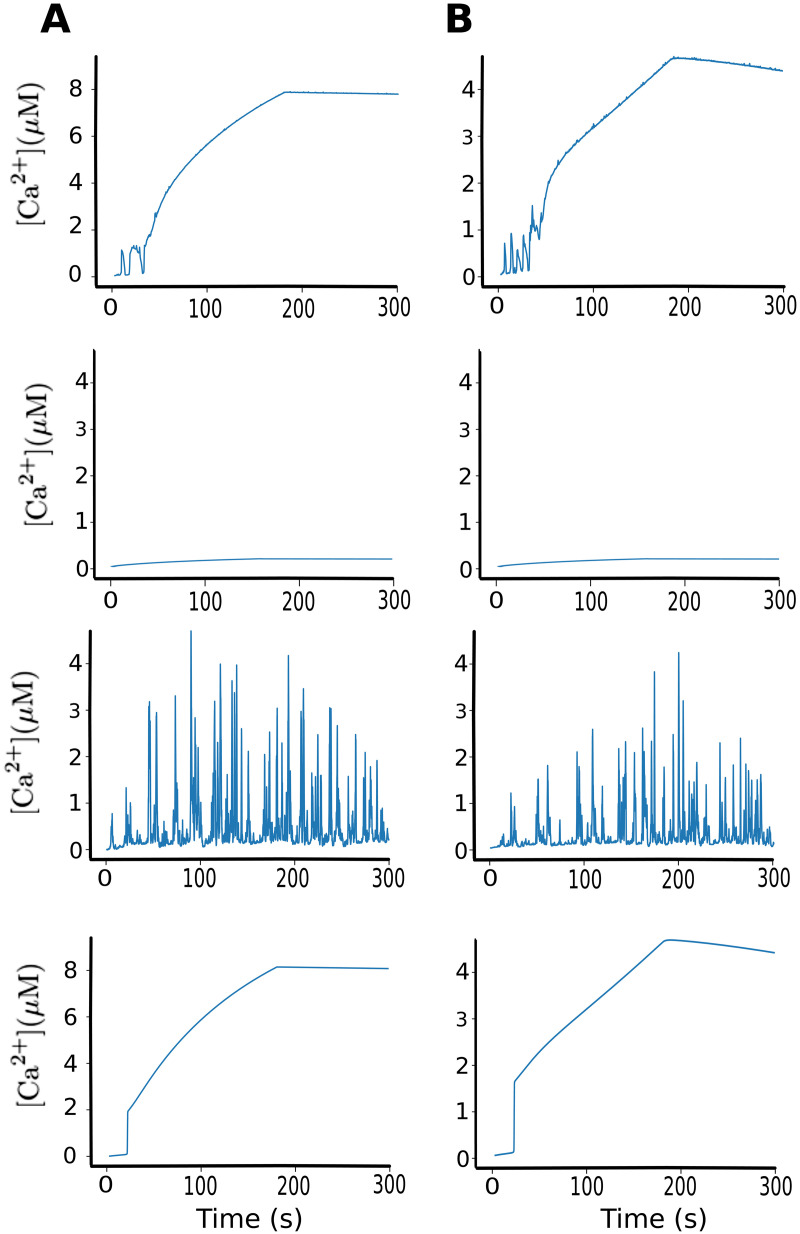
Effects of blocking different components of CICR on Ca^2^^+^ responses. Simulated OPC Ca^2+^ responses to 100 mM **(A)** ATP, and **(B)** glutamate stimulations under four conditions from top to bottom: in the presence of both IP3Rs and RyRs (WT), in the absence of RyRs and IP3Rs (RyR and IP3R KO; i.e., no CICR), in the absence of RyRs (RyR KO), and in the absence of IP3Rs (IP3R KO).

Interestingly, the entire Ca^2+^ response profiles (including the fast and slow components) associated with steep and shallow clusters associated with 100 mM ATP stimulation were consistent with the P2X7R current profiles induced by high ATP (or its agonist BzATP) stimulation observed in previous studies [28]. Indeed, it was previously shown that the kinetics P2X7R model (Fig 1F) alone is able to display two components in its current response upon high ATP stimualtion: an initial fast phase representing P2X7R channel opening followed by a slow rising phase representing P2X7R pore opening (or sensitization) [28–31]. Incorporating this kinetic model into the SSM successfully reproduced the two phases of Ca^2+^ responses (as shown for example in Fig 7A, top row), suggesting that Ca^2+^ signaling is shaped by receptor-generated currents. However, when CICR was blocked in these simulations (i.e., by inhibiting fluxes through IP3R and RyR), the biphasic response disappeared entirely (Fig 7A, second row), leaving only a slow and gradual rise in $[{\text{Ca}}^{2+}{]}_{\text{i}}$ following ATP stimulation. This indicates that while ATP-dependent Ca^2+^ responses are influenced by P2X7R kinetics, the synergy between the fast dynamics of CICR and P2X7R-mediated influx is essential for generating the observed dynamics. To further dissect which CICR Ca^2+^ flux is specifically contributing to this synergy, we then blocked either RyR flux (i.e., by “knocking out” RyR alone: RyR KO), or IP3R flux (i.e., by “knocking out” IP3R alone: IP3R KO) in the SSM. In the case of RyR KO (Fig 7A, third row), the two phases associated with Ca^2+^ responses seen in the WT case disappear leaving behind the VGCC-facilitated SCaLTs modulated by ATP. Conversely, in the IP3R KO case (Fig 7A, bottom row) VGCC-facilitated SCaLTs disappear as expected, whereas the two phases of ATP-induced Ca^2+^ responses remain intact, suggesting that RyR is the CICR component providing the synergy to produce the stereotypical ATP-mediated Ca^2+^ responses. This further underscores the necessity of the fast component of RyR flux dynamics for allowing evoked Ca^2+^ responses generated P2X7Rs to be shaped by its kinetics.

An unexpected observation arose from the IP3R KO simulations. In the absence of this flux (Fig 7A, second row), SCalTs disappeared completely, while evoked Ca^2+^ responses induced by ATP remained. Surprisingly, this closely resembles experimental recordings obtained with a steep gradient in the presence of Hanks’ Balanced Salt Solution. This observation suggests that this solution may be blocking IP3R-mediated flux and disrupting the clustering of these receptors. Confirmation of this hypothesis remains to be tested experimentally.

A similar pattern emerged when simulating the SSM response to 100 mM glutamate stimulation. In the presence of CICR (Fig 7B, top row), glutamate produced the expected Ca^2+^ response profile. However, when CICR was blocked (Fig 7B, second row), the evoked response again became a slow, gradual rise, mirroring the ATP case and reinforcing that CICR is required for AMPARs and NMDARs to generate responses consistent with their kinetics. Further examination of the individual contributions of IP3R and RyR fluxes showed that RyRs were the primary drivers of the synergistic evoked response (compare the third and fourth rows of Fig 7B).

### 3.6 Ca^2+^ responses to *in vivo*-like stimulation with ATP and glutamate

The prolonged ATP and glutamate stimulations were performed *in vitro*, but such sustained stimulations do not occur *in vivo*. Instead, *in vivo* stimulations are more likely to be briefer and occur in a more stochastic manner, with varying amplitudes over time. It would therefore be interesting to investigate how Ca^2+^ signals respond to randomly timed ATP and glutamate stimulations, better reflecting physiologically-relevant *in vivo* conditions.

**Fig 8:**
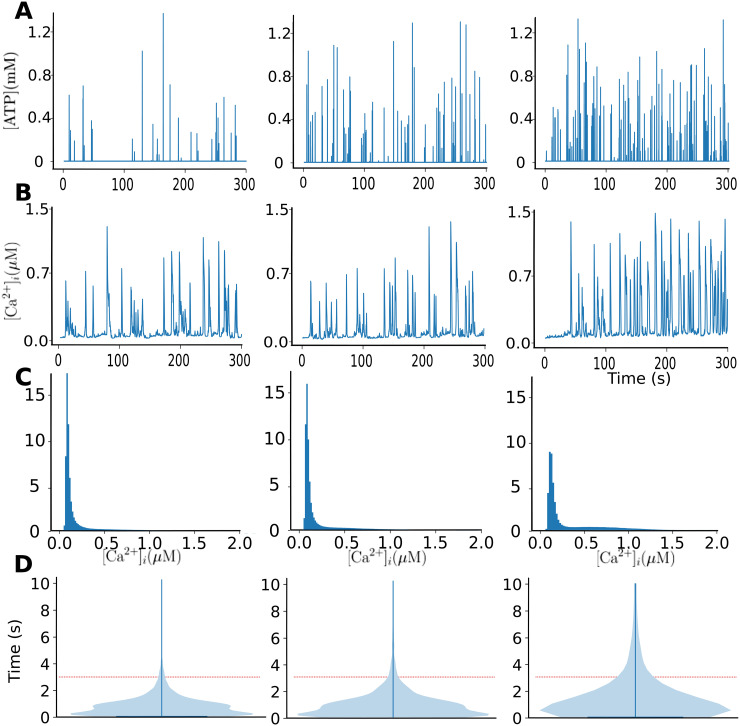
Simulated Ca^2^^+^ responses to random ATP stimulations mimicking *in vivo* conditions. **(A)** The random events of ATP stimulations in the form of brief 0.1 s pulses with varying amplitudes and frequencies corresponding to low (0.1 Hz; left), medium (0.25 Hz; middle) and high (0.5 Hz; right) stimulation rates. **(B)** Ca^2+^ transients (or spikes) combining both SCaLTs and evoked responses induced by the random ATP stimulations described in panel A, respectively. **(C)** Distributions of Ca^2+^ signals from 50 simulations of each of the three types of random stimulations shown in panel A, respectively, showing a significant increase in the amplitude and number of Ca^2+^ spikes. **(D)** Violin plots depicting the distribution of Ca^2+^ spike widths from 50 simulations of each of the three types of random stimulation shown in panel A, respectively. Red-dotted line separates wide spikes from narrow ones. Notice how an increase in the frequency of ATP stimulation leads to a moderate increase in the number of wide spikes.

**Fig 9:**
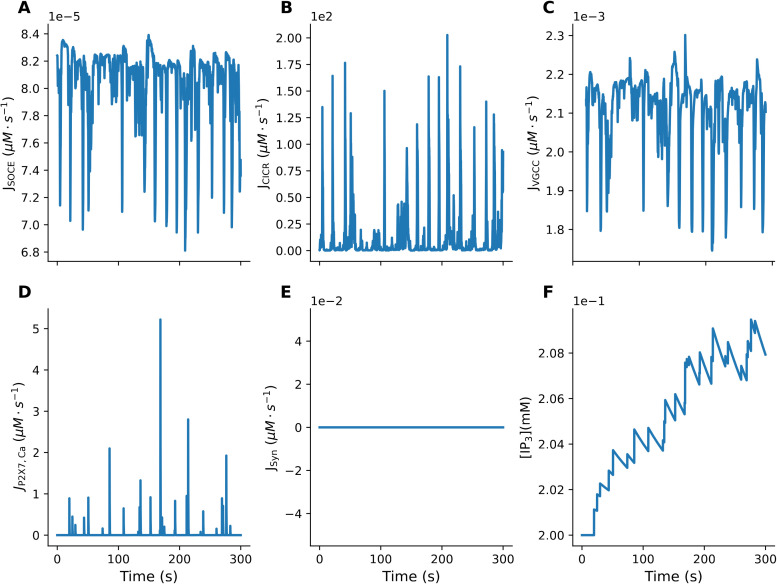
Simulated Ca^2^^+^ fluxes under random ATP stimulations mimicking *in vivo* conditions. Simulated Ca^2+^ flux through **(A)** store-operated Ca^2+^ entry ($J_{SOCE}$), **(B)** CICR ($J_{CICR}=J_{IP3}+J_{Ry}$), **(C)** VGCC ($J_{VGCC}=J_{L-type}+J_{T-type}$), **(D)** P2X7Rs ($J_{P2X7,Ca}$), and **(E)** AMPARs and NMDARs ($J_{Syn}=J_{AMPA,Ca}+J_{NMDA,Ca}$), along with (F) simulated cytosolic IP3 concentration ($[IP3{]}_{i}$).

To address this, we first applied random ATP stimulations (Fig 8A) in the form of brief 0.1 s pulses of varying amplitudes to the SSM and quantified their effects on Ca^2+^ spiking (Fig 8B) and fluxes (Fig 9). The ATP pulses were delivered randomly at low (left), intermediate (middle), and high (right) frequencies (Fig 8A). The resulting steady-state Ca^2+^ signals were then evaluated using two metrics: (i) the signal distribution, obtained by binning and averaging 50 simulated traces (Fig 8C), and (ii) the full width at half-maximum of Ca^2+^ spikes encompassing both SCaLTs and evoked responses (Fig 8D).

Our results showed that random, brief ATP stimulations generate broader waves (S6A Fig) than those seen in the absence of ATP (Fig 4A), indicating a gradual shift toward elevated intracellular Ca^2+^ levels (Fig 8C) and an increase in the proportion of wider Ca^2+^ spikes (Fig 8D), especially when increasing the stimulation frequency. Indeed, more frequent ATP applications lead to prolonged elevations in Ca^2+^ signals, particularly when compared to control conditions. Evaluating how the various fluxes respond to high-frequency ATP stimulation, we found that CICR fluxes (via both IP3Rs and RyRs) are markedly increased (Fig 9B) relative to when there is no ATP stimulations (S5 Fig). This enhancement is driven primarily by Ca^2+^ influx through P2X7Rs (Fig 9D) and by a rise in $[IP3{]}_{i}$ above its potentiation threshold (defined by the parameter *d*_1_) for IP3Rs (Fig 9F). The pronounced CICR flux is accompanied by an increase in SOCE (Fig 9A), to prevent ER depletion, but no significant change in VGCC-mediated Ca^2+^ influx (Fig 9C) compared to no ATP stimulation (S5 Fig).

**Fig 10:**
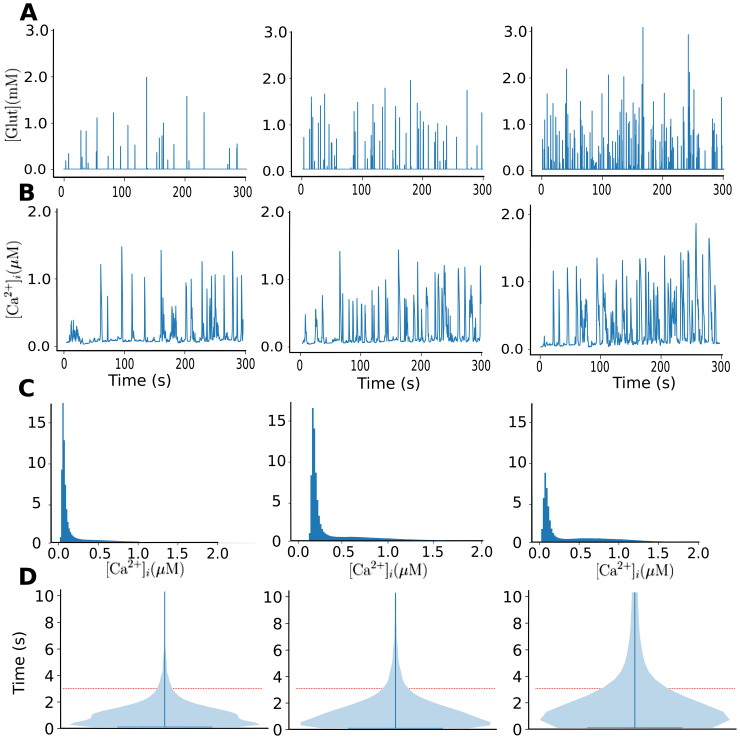
Simulated Ca^2^^+^ responses to random glutamate stimulations mimicking *in vivo* conditions. **(A)** The random events of glutamate stimulations in the form of brief 0.1 s pulses with varying amplitudes and frequencies corresponding to low (0.1 Hz; left), medium (0.25 Hz; middle) and high (0.5 Hz; right) stimulation rates. **(B)** Ca^2+^ transients (or spikes) combining both SCaLTs and evoked responses induced by the random glutamate stimulations described in panel A, respectively. **(C)** Distributions of Ca^2+^ signals from 50 simulations of each of the three types of random stimulations shown in panel A, respectively, showing a moderate increase in the number of spikes with large amplitudes without significant change in amplitude. **(D)** Violin plots depicting the distribution of Ca^2+^ spike widths from 50 simulations of each of the three types of random stimulation shown in panel A, respectively. Red-dotted line separates wide spikes from narrow ones. Notice how an increase in the frequency of glutamate stimulation leads to both a significant increase in the width of Ca^2+^ spikes as well as the number of such spikes.

**Fig 11:**
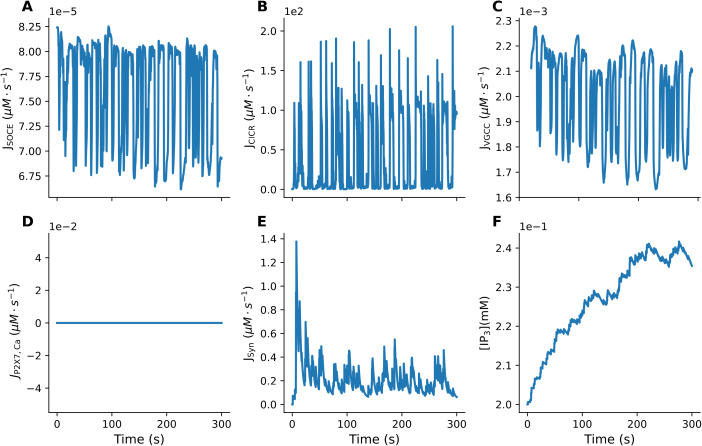
Simulated Ca^2^^+^ fluxes under random glutamate stimulations mimicking *in vivo* conditions. Simulated Ca^2+^ flux through **(A)** store-operated Ca^2+^ entry ($J_{SOCE}$), **(B)** CICR ($J_{CICR}=J_{IP3}+J_{Ry}$), **(C)** VGCC ($J_{VGCC}=J_{L-type}+J_{T-type}$), **(D)** P2X7Rs ($J_{P2X7,Ca}$), and **(E)** AMPARs and NMDARs ($J_{Syn}=J_{AMPA,Ca}+J_{NMDA,Ca}$), along with **(F)** simulated cytosolic IP3 concentration ($[IP3{]}_{i}$).

Although random, brief glutamate stimulations at low, intermediate and high frequencies induce very similar effects to Ca^2+^ spiking and fluxes to those seen with ATP stimulations (compare Figs 8 and 10, as well as Figs 9 and 11), glutamate stimulations have a more pronounced impact, producing wider spikes especially at intermediate to high frequencies, in agreement with the dynamics of the DTM observed when analyzing its bifurcation structure (Fig 3); this suggests that OPCs are more sensitive to glutamate than ATP (Figs 8D and 10D). Spatiotemporally, these prolonged elevations in Ca^2+^ were very pronounced during glutamate stimulations compared to ATP stimulations (S6 Fig) at intermediate and high frequencies. It is important to note that in both cases, the fluctuations in membrane voltage were not significantly altered by the frequency of these stimulations, except for the slight hyperpolarization seen in the membrane potential at high frequency stimulations (S7 Fig), affecting VGCC-mediated Ca^2+^ influx (Fig 11C).

Together, these findings indicate that ATP and especially glutamate stimulations mimicking *in vivo* conditions can induce broader Ca^2+^ spikes in OPCs; this may have significant implications on the functional role of OPCs and their maturation into oligodendrocytes.

## 4 Discussion

Using a computational modeling approach, we investigated in this study how spontaneous and evoked Ca^2+^ responses due to stimulations with ATP and glutamate affect Ca^2+^ transients in OPCs. We expanded our previously developed spatiotemporal stochastic flux-balance Ca^2+^ model [12] to incorporate not only the dynamics of membrane voltage, modeled using the Hodgkin-Huxley formalism and regulated by L-type and T-type Ca^2+^ channels, but also the kinetics of both P2X7Rs and glutamatergic AMPARs and NMDARs (all assumed uniformly expressed along the length of the cell model). Because these latter receptors are not specific, we also tracked through the model the changes in Na^+^ and K^+^ concentrations ($[{\text{Na}}^{+}{]}_{\text{i}}$ and $[{\text{K}}^{+}{]}_{\text{i}}$, respectively, assumed spatially homogeneous across the length of the cell). The resulting model both maintained the previously observed physiological and dynamic properties of the original model [12], as well as provided new insights into how VGCCs shape SCaLT dynamics and how purinergic and glutamatergic receptors drive evoked Ca^2+^ responses and modulate their spiking activity. We note that incorporating non-uniform receptor expression could meaningfully alter local Ca^2+^ dynamics, and exploring this assumption represents a promising direction for future work.

The two key components of the original model [12], including CICR via IP3Rs and RyRs and an Ornstein-Uhlenbeck noise process, enabled the model to previously generate random spiking events in the form of SCaLTs. In this study, the inclusion of VGCCs further amplified these random spiking events and increased their frequency. Previous research [7,8,10] has suggested that myelination of axonal fibers is Ca^2+^-dependent and that the frequency of Ca^2+^ spikes plays a crucial role in regulating this process. Specifically, Ca^2+^ spike frequency must fall within an optimal “Goldilocks zone” to promote effective myelination by oligodendrocytes. The increase in spiking frequency induced by L- and T-type Ca^2+^ channels may help shift the system into this optimal range, thereby enhancing myelination. Further analysis of the steady-state dynamics of the DTM, in the absence of noise and diffusion, showed that stimulation with ATP and glutamate can transition the system into an oscillatory (spiking) regime. This transition favors deterministic dynamics over the stochastic nature of SCaLTs, potentially allowing Ca^2+^ spiking to stabilize within the so-called ’Goldilocks zone’.

P2x7Rs are well known purinergic receptors that, unlike P2X2Rs and P2X4Rs, exhibit two current phases upon stimulation with high [ATP] (or its agonist BzATP) [28–31,56–58]: a fast component (previously labeled *I*_1_), followed by a slow one (previously labeled *I*_2_). It was shown in these studies that the fast component is mainly due to channel opening, while the slow one is due to receptor sensitization (or pore opening). Recording the associated Ca^2+^ transients in these cells upon these ATP stimulations showed that these evoked responses exhibit identical profiles with a fast rise (corresponding to *I*_1_) followed by a slow rise (corresponding to *I*_2_) in a manner similar to recordings shown in this study. By excluding CICR in the SSM, this two-phase profile disappeared completely, and only a very slow rise in $[{\text{Ca}}^{2+}{]}_{\text{i}}$ was observed. In this study, we highlighted the role of CICR in this process and emphasized that it extends also to glutamatergic receptors, including NMDARs and AMPARs.

In our simulations, we retained SCaLT activity when analyzing Ca^2+^ responses to prolonged ATP and glutamate stimulations, as the specific effects of Hanks’ Balanced Salt Solution are not fully understood. Because the experiments presented in this study are dominated by P2X7 and glutamatergic receptor activity, we expected any influence of Hanks’ Balanced Salt Solution to be minor and the model to adequately capture the key features of Ca^2+^ dynamics. However, through our subsequent analysis of the SSM, we discovered that this solution likely attenuates flux through IP3Rs by disrupting their opening–closing dynamics and receptor clustering. This interpretation is in agreement with the role of IP3Rs as the primary initiators of stochastic Ca^2+^ events in our framework, whereas RyRs primarily serve to amplify these events through CICR [59]. Consistent with this interpretation, our analysis showed that RyR flux, rather than IP3R flux, is required for P2X7- and glutamatergic-receptor–mediated Ca^2+^ entry to generate their three stereotypical response profiles (Fig 7). In this context, suppressing IP3R activity effectively removes the dominant source of stochastic fluctuations, leading to a reduction in spontaneous calcium transients, while RyR-mediated amplification remains intact. Notably, stochastic noise in the model was incorporated into the inactivation variable of the IP3R flux; when this flux is blocked, these stochastic fluctuations are removed, and RyR flux alone enables P2X7 and glutamatergic receptors to reproduce the observed response profiles without SCaLTs, closely matching the experimental traces. Such distinction mirrors our knockout analyses, where IP3R suppression eliminates the initiation of calcium events, whereas RyR suppression preserves small, localized fluctuations but prevents their amplification into larger transients. This observation suggests that Hanks’ Balanced Salt Solution may act, at least in part, by blocking IP3Rs, thereby reducing stochastic fluctuations while leaving RyR-mediated amplification intact. Accordingly, our model predicts that bathing cells in Hanks’ Balanced Salt Solution promotes more quiescent calcium signals during prolonged neurotransmitter stimulation by suppressing IP3R-driven noise. These findings remain to be validated experimentally.

Active axons communicate with local oligodendrocytes through two primary mechanisms: the vesicular release of glutamate and the non-vesicular release of intracellular ATP [60]. ATP is released via a volume-regulated anion channel, which is activated by osmotic swelling resulting from ion influx during action potentials [61]. Similarly, glutamate is released in an activity-dependent manner in both developing and mature oligodendrocytes [62]. Both ATP and glutamate regulate Ca^2+^ dynamics in myelinating oligodendrocytes, influencing myelin formation. Variations in the release patterns of these molecules along a single axon may contribute to the heterogeneous myelin distribution observed in the human cortex [63]. Approximately half of the Ca^2+^ transients in developing oligodendrocytes are driven by axonal action potentials, but the remaining ones occur independently of action potentials [10]. Interestingly, the positive correlation between the frequency of Ca^2+^ transients and speed of myelin sheath elongation is thought to be linked to the role of Ca^2+^ in actin polymerization in the myelin sheath [8]. Additionally, it has been experimentally demonstrated that these oscillations are activity-dependent, although the underlying mechanism remains unclear. In this study, we examined how applying random ATP and glutamate stimulation events mimicking *in vivo*-like conditions affect Ca^2+^ transients in the SSM. Our goal was to explore how Ca^2+^ spikes are formed as a combination of both evoked ATP- and glutamate-dependent responses and spontaneous events that are enhanced by VGCCs. Our results showed that even at low ATP and glutamate stimulation frequencies, the SSM generated broader Ca^2+^ spikes, which may serve a distinct role from narrower spikes, such as in cell differentiation. In contrast, the amplitude of the Ca^2+^ spikes remained largely unchanged. While this stability in amplitude aligns with previous studies suggesting that Ca^2+^ spike amplitude and shape are relatively insensitive to stimulation strength [64–66], the observed increase in spike width does not. This discrepancy is likely due to the stochastic nature of ATP and glutamate stimulation interacting with an excitable system, leading to random spiking events. More specifically, ATP and glutamate stimulation appear to drive the system from a spiking regime into an overstimulated regime [67], operating near the boundary between the two. Interestingly, these overstimulated responses arise despite the fact that ATP and glutamate stimulation events are brief (0.1 s), which results in relatively small $J_{SOCE}$ and modest changes in [IP3]_*i*_. This further emphasizes that the observed dynamics are governed by the excitable nature of the system, whereby even weak, transient inputs can drive transitions between dynamical regimes.

Stretch-activated Ca^2+^ channels (SACs) may contribute to Ca^2+^ influx in oligodendrocytes, particularly within the soma. These mechanosensitive channels are known to play a crucial role in cellular motility [68] and have been implicated in mechano-electric feedback mechanisms that regulate Ca^2+^ transients in cardiomyocytes [69]. In oligodendrocytes, osmotic swelling caused by Ca^2+^ influx could activate SACs, further amplifying Ca^2+^ currents. This feedback loop may play a role in regulating intracellular Ca^2+^ signaling and volume homeostasis in response to mechanical or osmotic changes. The role of SAC was not incorporated into the SSM but could be a promising avenue for exploring its impact on Ca^2+^ spikes.

Given that myelination in the central nervous system is closely linked to the neural activity of neighboring cells, our flux-balance-based SSM provided a framework for investigating the underlying dynamics of Ca^2+^ signals in OPCs. This approach offered fresh insights into the fundamental Ca^2+^ transients that drive neuro-oligodendrocyte signaling. Ultimately, the development of the SSM represents a crucial step toward unraveling the complexities of neuro-oligodendrocyte interactions, with potential implications for advancing our understanding of myelin-related processes.

## Supporting information

S1 Fig Effects of key Ca^2+^ fluxes on voltage-independent (or intrinsic) SCaLTs when the voltage, P2X7Rs, AMPARs and NMDARs are absent. As in [12], blocking **(A)** IP3Rs produces quiescent signal, **(B)** SERCA pumps produces one single Ca^2+^ transient, **(C)** RyRs produces reduced number of SCaLTs, and **(D)** mode 1, mode 2 and both modes of NCX produces a decrease, an increase and no effect, respectively. Insets in C and D are the average number of SCaLTs of 50 simulations for each condition.
(EPS)

S2 Fig Illustration of the stimulation protocol. Random spike-train stimulations were applied uniformly across the entire cell (modeled as a one-dimensional domain of length L) using **(A)** ATP and **(B)** glutamate, delivered as repetitive square-wave pulses with varying frequency and amplitude (as shown in the magnified insets of each panel). High-frequency stimulations are presented for illustrative purposes.
(EPS)

S3 Fig Representative steady state time-series simulations illustrating model responses to ATP. Ca^2+^ spikes generated from simulations initialized within **(A)** the regular spiking regime located between the two torus bifurcations: TR1 and TR2, and **(A)** the quiescent regime to the left of the left of Hopf bifurcation.
(EPS)

S4 Fig Representative steady state time-series simulations illustrating model responses to glutamate. Ca^2+^ spikes generated from simulations initialized within **(A)** the mixed-mode oscillation regime between the torus bifurcation: TR and period doubling bifurcation: PD, **(B)** regular spiking regime to the right of PD and far from the homoclinic bifurcation, and **(C)** regular spiking regime near the homoclinic bifurcation.
(EPS)

S5 FigSimulated wild-type (WT) OPC fluxes in the absence of neurotransmitters.Simulated Ca^2+^ flux through **(A)** store-operated Ca^2+^ entry ($J_{SOCE}$), **(B)** CICR ($J_{CICR}=J_{IP3}+J_{Ry}$), **(C)** VGCC ($J_{VGCC}=J_{L-type}+J_{T-type}$), **(D)** P2X7Rs ($J_{P2X7,Ca}$), and **(E)** AMPARs and NMDARs ($J_{Syn}=J_{AMPA,Ca}+J_{NMDA,Ca}$), along with **(F)** simulated cytosolic IP3 concentration, taken as constant ($[IP3{]}_{i}=0.2$).(TIFF)

S6 FigSpatiotemporal dynamics of cytosolic Ca^2+^ concentration in WT OPCs subjected to stochastic neurotransmitter stimulation at varying frequencies.Heatmaps of Ca^2+^ concentration ($[{\text{Ca}}^{2+}{]}_{\text{i}}$) along the entire length of the cell *L* = 8 μm in response to **(A)** ATP, and **(B)** glutamate stimulation, color-coded according to the color scale on the right of each panel. For both ATP and glutamate, stimulation is applied at low (left), intermediate (middle) and high (right) frequencies. The transient Ca^2+^ events in the form of waves are triggered by the combination of SCaLTs and evoked responses occurring along the spatial domain during a 300-second simulation.(EPS)

S7 Fig Fluctuations in membrane voltage upon random stimulations with ATP (top row) and glutamate (bottom row), mimicking *in vivo* conditions. Random stimulations are done at **(A,D)** low, **(B, E)** intermediate, and **(C,F)** high frequencies.
(EPS)
