# Supplementary figures and images for: Mechanisms underlying spontaneous and evoked calcium responses in oligodendrocyte precursor cells: A modeling investigation
Source: PLoS Comput Biol. 2026 Jun 18;22(6):e1013430. doi: 10.1371/journal.pcbi.1013430 (PMC13318057; doi:10.1371/journal.pcbi.1013430)

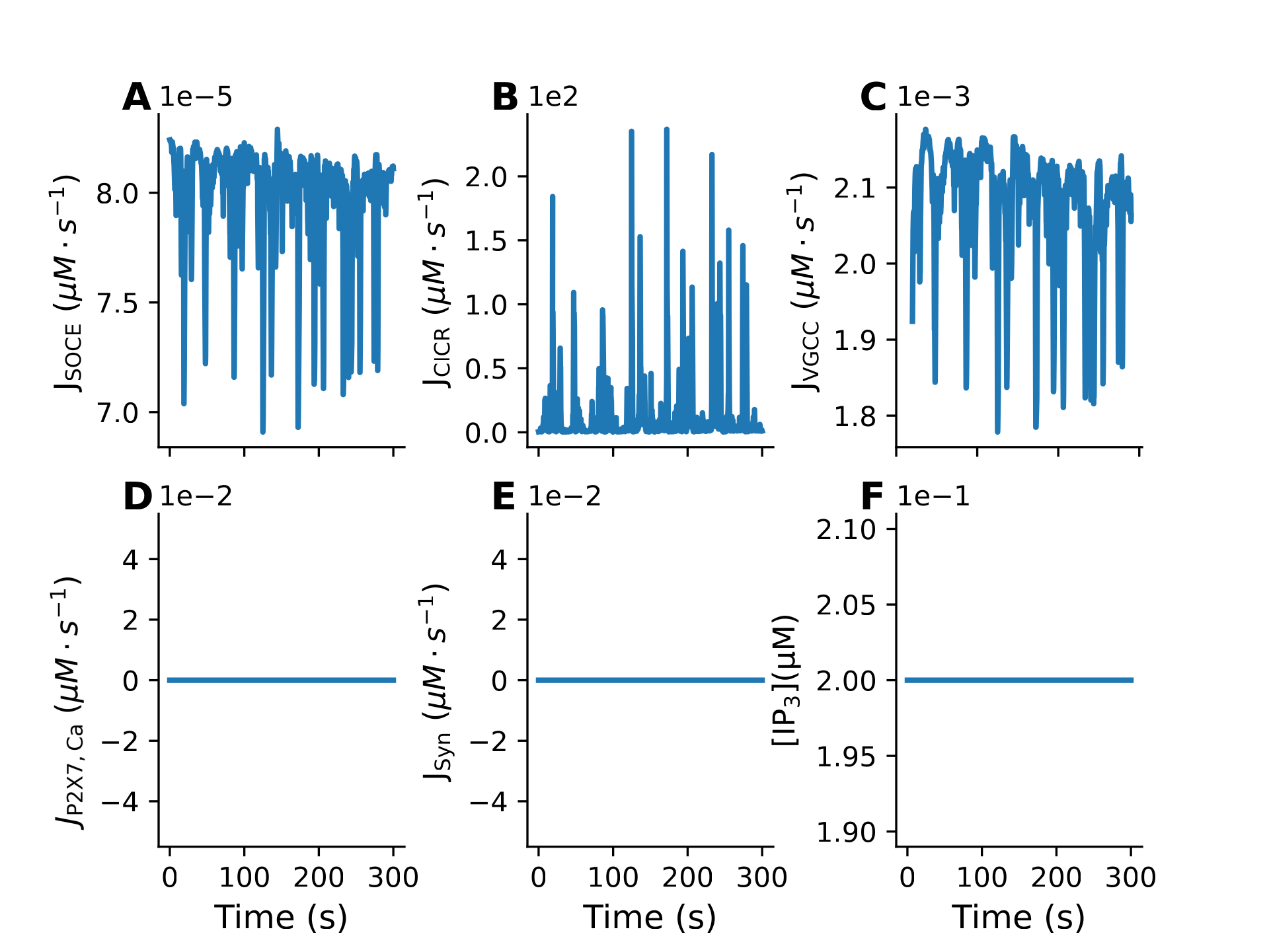

Supplement: S5 Fig — Simulated Ca2+ flux through (A) store-operated Ca2+ entry (JSOCE), (B) CICR (JCICR=JIP3+JRy), (C) VGCC (JVGCC=JL−type+JT−type), (D) P2X7Rs (JP2X7,Ca), and (E) AMPARs and NMDARs (JSyn=JAMPA,Ca+JNMDA,Ca), along with (F) simulated cytosolic IP3 concentration, taken as constant ([IP3]i=0.2). (TIFF) [file pcbi.1013430.s005.tiff]
